# Improvements at the Westminster General Dispensary

**Published:** 1905-01-28

**Authors:** 


### IMPROVEMENTS AT THE WESTMINSTER GENERAL DISPENSARY.

The Westminster General Dispensary, Gerrard Street, Soho, one of the oldest dispensaries in London, has recently undergone great alterations and improvements, through the generosity of Mr. Blackwell. The whole building is now lit by electricity which includes portable electric lamps, and heated throughout, and the walls have all been painted white. On the ground floor the surgical room has been fitted with a new marble terrazzo pavement. There are two new Doulton basins with three-way taps, and some Bailey's glass and enamel dressing and instrument cases. Glass-doors have been fixed to the cupboards, and shelves placed on the window-ledges and mantelpiece. In the dispensary itself there are new cupboards, and a sink removed from a dark corner to a place under the window has now a hot-water tap. Improvements have also been made in Dr. Gilbert's (the resident medical officer) quarters; and, altogether, although the old house retains its fine staircase, quaint old benches and other antiquarian interests, the modern sanitary and surgical improvements recently added make it an infinitely better dispensary.

